# Tumor microbiome – an integral part of the tumor microenvironment

**DOI:** 10.3389/fonc.2022.1063100

**Published:** 2022-11-24

**Authors:** Sona Ciernikova, Aneta Sevcikova, Viola Stevurkova, Michal Mego

**Affiliations:** ^1^ Department of Genetics, Cancer Research Institute, Biomedical Research Center of Slovak Academy of Sciences, Bratislava, Slovakia; ^2^ 2nd Department of Oncology, Faculty of Medicine, Comenius University, Bratislava and National Cancer Institute, Bratislava, Slovakia

**Keywords:** gut microbiota, cancer, tumor microenvironment, tumor microbiome, bacterial diversity, treatment response

## Abstract

The tumor microenvironment (TME) plays a significant role in tumor progression and cancer cell survival. Besides malignant cells and non-malignant components, including immune cells, elements of the extracellular matrix, stromal cells, and endothelial cells, the tumor microbiome is considered to be an integral part of the TME. Mounting evidence from preclinical and clinical studies evaluated the presence of tumor type-specific intratumoral bacteria. Differences in microbiome composition between cancerous tissues and benign controls suggest the importance of the microbiome-based approach. Complex host-microbiota crosstalk within the TME affects tumor cell biology *via* the regulation of oncogenic pathways, immune response modulation, and interaction with microbiota-derived metabolites. Significantly, the involvement of tumor-associated microbiota in cancer drug metabolism highlights the therapeutic implications. This review aims to summarize current knowledge about the emerging role of tumor microbiome in various types of solid malignancies. The clinical utility of tumor microbiome in cancer progression and treatment is also discussed. Moreover, we provide an overview of clinical trials evaluating the role of tumor microbiome in cancer patients. The research focusing on the communication between the gut and tumor microbiomes may bring new opportunities for targeting the microbiome to increase the efficacy of cancer treatment and improve patient outcomes.

## 1 Introduction

A variability of microorganism resident in the human body, collectively termed the microbiota, showed playing a critical role in human health and disease. The enormous development of next-generation sequencing technologies and bioinformatic tools enabled accessing the profound impact of microbiomes - the ecosystems created by resident microbes, their genomes, and functional interactions, on cancer development and treatment. Cancer researchers and clinicians aim to uncover the underlying mechanisms in host-microbiota crosstalk, leading to the clinical utility of the microbiome-based approach. A significant impact of the gut microbiome on the host physiology has been described mainly *via* modulation of the host immune system, specific function in host metabolism, production of microbiota-derived metabolites, and protection against pathogens (1, 2). Changes in the gut microbiome composition related to dysbiosis contribute to severe pathological conditions, including cancer (3, 4). However, the clinical relevance of the organ-specific microbiomes remains unexplored. The studies showed that infectious microorganisms play a role in approximately 20% of human cancers (5, 6). The existence of specific blood and tissue microbial signatures in different malignancies has been uncovered by reexamination of whole genome and transcriptome sequencing studies (7). Recently, the microbial analysis of tumor samples from seven different malignancies (breast, lung, ovary, pancreas, melanoma, bone, and brain cancer) described the tumor type-specific bacteria localized in cancer and immune cells (8).

Since mounting evidence from animal models and clinical studies, several prospective new hallmarks and enabling characteristics have been added to a very well-known comprehensive concept on the hallmarks of cancer. Besides phenotypic plasticity, non-mutational epigenetic reprogramming, senescent cells, also polymorphic microbiomes became incorporated as core components of the hallmarks of cancer conceptualization (9). Research using fecal transfer from cancer patients to mouse models suggested the existence of both cancer-protective and tumor-promoting microbiomes containing particular bacterial taxa that are capable to modulate the tumorigenic and cancer progression pathways, and have an impact on treatment efficacy (10–12).

Recent advances in cancer research highlight that not only genetic and epigenetic alterations in cancer cells, but also dynamic interactions between all components within the tumor microenvironment (TME) have strong associations with tumor development. Apart from complex signaling communication between cellular and non-cellular TME elements, mutual interactions with microbial components showed a considerable effect on tumorigenesis and tumor progression in a local manner. Mounting evidence suggests the direct involvement of the tumor-associated microbiota in oncogenesis and tumor physiology by the activation of oncogenic pathways, promotion of mucosal inflammation, or metabolic and immune dysregulation. Furthermore, the relationship between the gut/tumor microbiome and response to cancer treatment and drug metabolisms has been comprehensively reviewed (13).

In contrast to gastrointestinal cancers, limited evidence exists about the link between the composition of intratumoral microbiota and other malignancies. Herein, we review current data focusing on the role of the tumor microbiome, as an integral part of the TME. The studies of microbial communities within different solid malignancies are discussed. We provide an overview of the clinical trials, evaluating the presence of microbial communities within tumor tissue samples. Moreover, the clinical relevance and possible therapeutic implications of tumor microbiome analysis in disease progression and cancer treatment efficacy will be outlined.

## 2 The composition of the tumor microenvironment

The composition of the TME differs between tumor types. However, in general, TME consists of proliferating malignant cells and non-malignant components including immune cells such as microglia, macrophages, and lymphocytes, elements of the extracellular matrix (ECM), stromal cells, and endothelial cells (14). The TME contributes to the promotion of angiogenesis, aiming to overcome hypoxic conditions and restore supplementation with oxygen and nutrients (15). Tumors interact with the surrounding microenvironment and other host organs through the blood or lymphatic circulatory system (16).

Immune cells, important TME constituents affecting cancer growth and progression (17), can be divided into two specific categories; innate immune cell types (dendritic cells, innate lymphoid cells, macrophages, myeloid-derived suppressor cells, natural killer cells, neutrophils) and adaptive immune cell types (B and T cells). The presence of infiltrated innate and adaptive cells within tumors can support either the anti or pro-tumorigenic process (18). The ECM is a highly dynamic non-cellular component of the TME, predominantly composed of collagen, fibronectin, elastin, and laminin (15). A key role of ECM is to direct both cell migration and proliferation (19) and can affect the track and speed of cell migration *via* its topography and physical properties. According to the findings, tumor cells, as well as cancer-associated fibroblasts (CAFs) are the main source of ECM molecules (20, 21). In many solid tumors, ECM forms almost 60% of the tumor mass and changes during cancer progression and metastasis (22). Stromal cells, including CAFs, create the majority of tumor stroma and promote not only tumor initiation but also angiogenesis and cancer progression. Studies confirmed that CAFs contribute to therapeutic resistance in breast cancer (23). Lakins et al. showed that CAFs directly suppress anti-tumor T-cells within the TME (24). The tumor vasculature is irregular and chaotic in contrast to the normal vasculature of arranged and differentiated arteries, arterioles, capillaries, venules, and veins. Blood vessels are more represented at the tumor interface with decreased vascularity towards the central region, leading to zones of ischemia and necrotic area (25). The vascular density decreased with the tumor growth, which consequently leads to tumor necrosis. The unbalanced secretion of proangiogenic vascular endothelial growth factor-A (VEGF-A) results in the formation of blood vessels (26, 27).

Cancer cell proliferation and metastatic spreading are associated with reprogramming the TME, aiming to support the supplementation of tumor cells with nutrients (28). Complex interactions are determined by the structural and biochemical properties, and also by mutual communication (29). Cross-talk within the TME is mediated by the mixture of cytokines, chemokines, growth factors, and inflammatory mediators together with matrix remodeling enzymes. Moreover, novel ways of communication *via* extracellular vesicles (EV) including exosomes and apoptotic bodies as well as exosome-derived miRNAs are being explored (30, 31).

**Figure 1:**
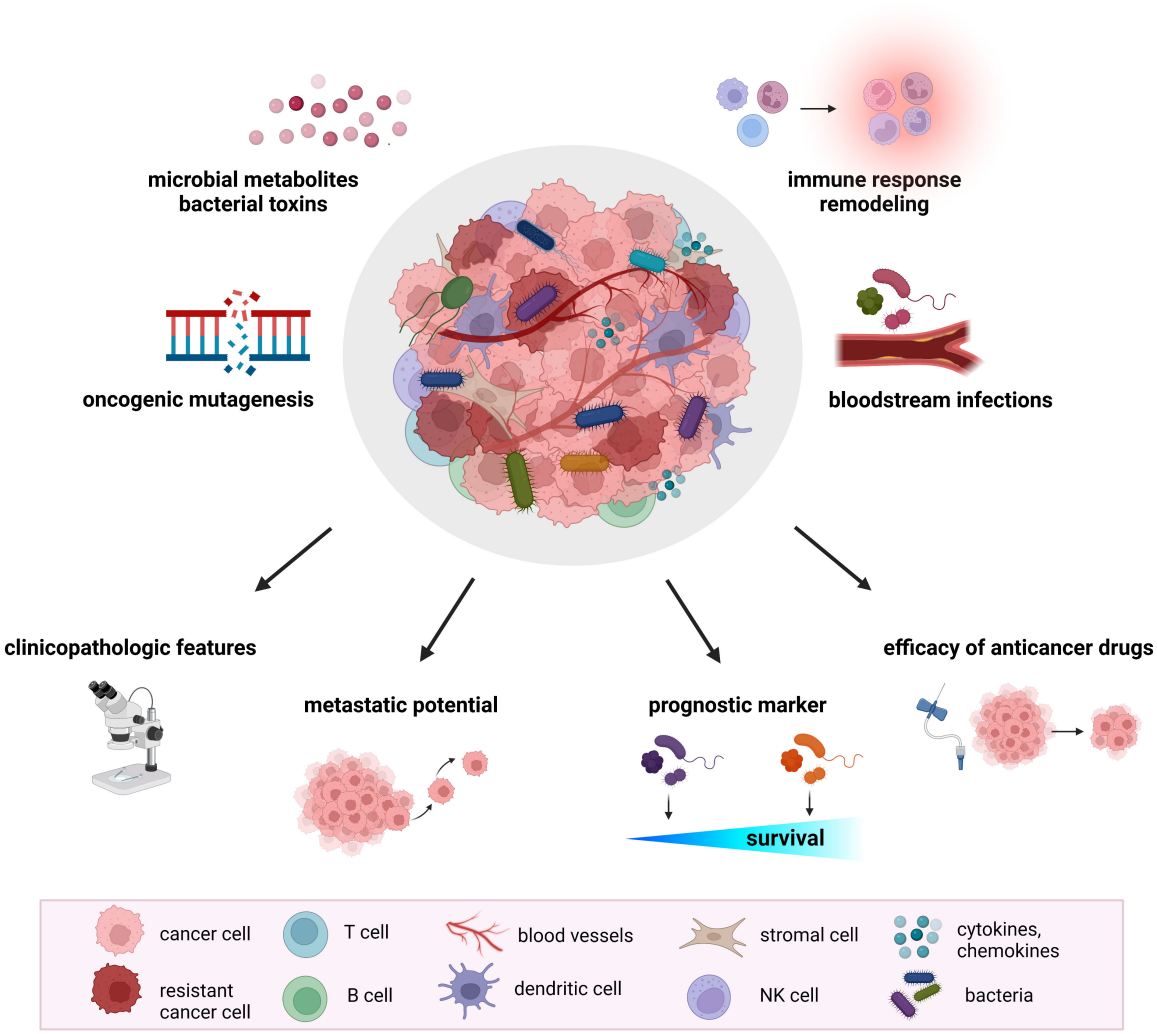
The role of tumor microbiome in cancer development and treatment. Proposed mechanisms by which the tumor microbiome affects tumorigenesis, cancer progression, and response to therapeutic agents include increased mutagenesis, regulation of oncogenes and oncogenic pathways, modulation of host immune response pathways, cancer drug metabolism, production of bacterial toxins and microbiota-derived metabolites. Mounting evidence from animal models and clinical studies revealed the association of tumor microbiome with clinicopathologic features, cancer treatment efficacy, metastatic potential, and cancer survival.

Recent research documented a significant impact of the host gut microbiome on shaping the TME. The microbiome regulates the immune and hormonal signaling, having an impact on components of the TME due to the modulation of the processes included in tumor-promoting. In addition, microbiota-derived metabolites can enter the TME *via* circulation and become a part of the microenvironment (32). Numerous studies confirmed the presence of tumor-type specific bacteria within the TME, suggesting their involvement in several pathways related to tumorigenesis and cancer progression (**Figure 1**).

## 3 The study of tumor microbiome in various solid malignancies

The occurrence of bacteria in tumors was reported more than 100 years ago but it is still unclear if their presence is useful for tumors or bacteria themselves. The study on mucosa-associated bacteria documented the presence of intracellular *Escherichia coli* in the colonic mucosa of patients with colorectal adenoma and carcinoma compared to controls (33). More recently, Nejman et al. focused on the validation of bacteria in a large group of 1010 tumor samples and 516 normal adjacent tissues. The tumor microbiome was analyzed in seven solid tumor types (glioblastoma multiforme, breast, ovary, bone, pancreas, melanoma, and lung cancer). The study revealed bacterial lipopolysaccharide (LPS) and 16S rRNA in all tumor types. The microbiome of breast tumors was more diverse than the microbiome of other tumor types. In every breast tumor sample was detected 16.4 bacterial species while less than 9 bacterial species were presented in other tumor samples. Proteobacteria and Firmicutes were the most presented in all tumor types while the Actinobacteria phylum including *Corynebacteriaceae* and *Micrococcaceae* families dominated in non-gastrointestinal tumors (8).

The overview of ongoing clinical trials concerning the role of tumor microbiome in different cancer types is shown in **Table 1**.

**Table 1:** Evaluating the role of tumor microbiome in different cancer types.

Study	Study design	Disease	Purpose	Patients (n)	Inclusion criteria/intervention	Study status
**NCT03586297**	An observational prospective study	triple-negative breast cancer	To analyze the neoadjuvant chemotherapy-induced tumor immunosurveillance and determine if intratumoral microbial composition correlates with anticancer immune responses within tumors	49 adults (female)	Samples from histologically confirmed cancer patients with a 1.5 cm mass lesion or greater will be collected during pretreatment, mid-treatment, and after the completion of chemotherapy.	Recruiting
**NCT04223102**	An interventional (clinical trial), single-group open-label study	locally advanced rectal cancer	To reveal the correlations between the microbiome composition in biopsy specimens from cancer patients and treatment response	40 adults	Rectal biopsy of the tumors from patients will be obtained during a sigmoidoscopy. Patients will be treated with neoadjuvant chemoradiation.	Recruiting
**NCT05324553**	An interventional (clinical trial), non-randomized study	cancer	To determine how the composition of tumor specimens may improve outcomes for cancer patients and use collected biopsies for developing preclinical models	50 adults	Patients as candidates will be selected for surgical procedures to obtain tissue samples.	Recruiting
**NCT04425122**	An observational prospective study	esophageal cancer	To investigate the microbiome in oesophageal cancer tissues and normal non-cancerous esophageal mucosa	200 adults	Patients will be scheduled for a routine upper endoscopy to obtain a biopsy from esophageal cancer tissues.	Recruiting
**NCT03959410**	An observational prospective study	cervical cancer	To evaluate the role of the cervical bacterial microbiome in cervical cancer patients positive for HPV DNA test	100 adults (female)	Women in fertile age who are positive for HPV DNA will be included in the study.	Recruiting
**NCT04260295**	An observational cross-sectional study	lung cancer	To explore specific pathogen distribution within lung cancer microbiome *via* metagenomic sequencing of lung puncture biopsies	100 lung cancer and 200 non-lung cancer adults	Patients with lung shadow will be admitted to the respiratory department to obtain lung lesion tissues. Biopsies will be provided for histopathology and microbiome analysis.	Unknown
**NCT05193162**	An observational retrospective study	pancreatic cancer	To evaluate the microbiome in biopsies from pancreatic cancer *via* 16S rRNA sequencing and by immunohistochemical and immunofluorescence detection	480 children/adults	Patients enrolled in the study will help to provide a new theoretical basis for the diagnosis and treatment of cancer. For microbiome analysis, bacterial DNA was extracted from the paraffin lesion tissues obtained by surgery tissue sampling.	Recruiting
**NCT04772001**	An interventional (clinical trial), single-group open-label study	cervical cancer	To define the microbiome composition within the tumor microenvironment by analysis through multi-omics/bioinformatic technologies	53 adults (female)	Participants will be selected based on criteria in the protocol. Women will be treated with hydrochloride anlotinib combined with concurrent radiochemotherapy.	Recruiting
**NCT03216135**	An observational case-control prospective study	esophageal cancer	To determine the role of the esophageal microbiome in the development of esophageal adenocarcinoma by microbiome profiling of tumor samples	50 adults	Females/males with known or suspected Barrett’s Esophagus and/or esophageal cancer will be included. Tumor tissues and a control squamous epithelium will be analyzed from the same patient.	Unknown
**NCT03885648**	An observational case-control cross-sectional study	breast cancer	To investigate the presence of bacteria, archaea, fungi, and viruses in breast tumor tissues by a metagenomic approach	150 adults (female)	Patients with surgically intervened breast cancer stages I and II will be involved in the study.	Active, not recruiting
**NCT03454685**	An observational case-only study	non-small cell lung cancer	To reveal microbial composition within tumors which can be used as a biomarker of lung cancer	100 adults	Female and male non-small cell lung cancer stages I-IV and healthy controls will be included without any intervention during the study.	Unknown
**NCT04669860**	An observational case-only prospective study	renal cell carcinoma	To identify the bacterial and fungal communities in renal tumor biopsies	90 adults	Patients will be scheduled for surgical removal of a kidney.	Recruiting

HPV, Human papillomavirus. The table summarizes the list of ongoing clinical trials concerning the presence of microbial communities within tumor tissue samples (according to https://clinicaltrials.gov/ ).

### 3.1 Colorectal tumor microbiome

Colorectal cancer (CRC) represents the malignancy with the most evident connection between the microbiome and cancer development, based on more than 50 years of investigation (34). Thus, microbiota modifications consider useful as a potential preventive and therapeutic tool in CRC tumorigenesis (35).

A higher abundance of *Fusobacterium nucleatum* was reported in colorectal adenomas and carcinomas compared to control tissues (36, 37). Additionally, *Fusobacterium* positively correlated with metastatic spreading since 74% (29/39) of patients with enrichment of this bacterium developed lymph node metastases (37). Mima et al. also found an increased amount of *F. nucleatum* in colorectal carcinomas, stage II-IV (38). The microbial differences between samples obtained from colon tumor tissues and adjacent non-malignant mucosa found overexpression of *Slackia* and *Collinsella* with decreased levels of *Citrobacter, Shigella, Cronobacter, Kluyvera, Serratia*, and *Salmonella* spp. in carcinomas (39). The findings from a study performed by Burns et al. revealed an increased amount of pathogenic bacteria *Providencia* and *Fusobacterium* in colon tumor tissues. It is known that *Fusobacterium* is implicated in colorectal carcinogenesis while *Providencia* showed to play a role in infections (40, 41). Both bacterial taxa share similar phenotypic characteristics, including the ability to affect and damage colorectal tissues. Patient-matched normal and tumor colon tissue samples showed differences in the abundance of Firmicutes, Bacteroidetes, and Proteobacteria. In particular, Proteobacteria dominated, whereas Firmicutes and Bacteroidetes were depleted in colon tumors. Moreover, the tumor microbiome characterizes decreased amounts of *Lachnospiraceae, Ruminococcaceae, Faecalibacterium prausnitzii, Bacteroides, Rikenellaceae*, and *Bacteroides uniformis* (41).

Geng et al. detected a lower abundance of *Microbacterium* and *Anoxybacillus* together with *Roseburia* enrichment in tumor tissues of Chinese colorectal cancer patients (42). The composition of the tumor microbiome was significantly different from adjacent non-neoplastic tissues, with an increase in Firmicutes and Fusobacteria. Overall, a higher number of bacterial genera were observed in cancerous samples. *Lactococcus, Bacteroides, Fusobacterium, Prevotella*, and *Streptococcus* dominated in tumors while *Pseudomonas* in adjacent non-cancerous tissues. The results also showed that the microbiota of proximal colon tumors was similar to that of distal colon cancer. These similarities are partially caused by stool passing through the intestinal tract (43). The analysis of intracellular bacteria reported an increased abundance of intracellular *Escherichia coli* in adenoma and carcinoma samples compared to control tissues obtained during routine colonoscopy (44). In addition, *Klebsiella pneumoniae, Pseudomonas aeruginosa, Enterococcus faecalis*, and *Bacillus cereus* were presented in biopsies from patients with colorectal adenomas (44). More recently, lower amounts of *Lanchnospiraceae, Ruminococcaceae/Faecalibacterium* were identified in colorectal carcinomas compared to polyps, adenomas, or control samples. A significantly higher level of *Bacteroides/Prevotella* and *Bacteroides/Porphyromonas*, especially *Porphyromonas asaccharolytica*, was detected in cancerous tissue (45). *Fusobacterium* DNA was detected in 181 colorectal cancer liver metastases. A lower density of CD8+ cytotoxic T cells and a higher density of myeloid-derived suppressor cells were observed in *Fusobacterium*-positive colorectal cancer liver metastases (46).

Animal models and clinical studies reported the role of microbiota-derived metabolites in colorectal carcinogenesis. A decreased butyrate and increased amounts of lactate, glutamate, alanine, and succinate were found in fecal samples and tumor tissues in CRC patients. Cancer-associated changes affected the metabolic pathways, including glucose and glycolytic metabolism, glutaminolysis, the tricarboxylic acid (TCA) cycle, and SCFA metabolism. A lower amount of fecal acetate correlated with alterations in glucose and myo-inositol levels. Based on the results, acetate showed the diagnostic potential as a single fecal biomarker for colorectal cancer detection (47). An earlier study from Lin and colleagues documented a decreased acetate, propionate, butyrate, glucose, and glutamine, while elevated proline, succinate, isoleucine, leucine, valine, alanine, glutamate, dimethylglycine, and lactate in stool samples of stage I/II CRC patients compared to healthy participants. Metabolomics significantly differentiated between I/II stage CRC patients and later stages of the disease. Changes in metabolite abundance participate in the disruption of bacterial ecology, malabsorption of nutrients, and increased glycolysis and glutaminolysis (48). Yang et al. confirmed that microbial metabolites differed between high-fat diet-fed mice and control diet-fed mice with an intraperitoneal injection of azoxymethane to mimic CRC. Glycerophospholipids, including lysophosphatidylcholine and lysophosphatidic acid, were elevated in mice with a high-fat diet. According to the findings, *Alistipes* spp. were enriched in the gut microbiome, while probiotic *Parabacteroides distasonis* was absent in high-fat-diet-fed mice. Antibiotic depletion of the gut microbiome retarded tumor formation in the high-fat diet-fed mice with decreased lysophosphatidylcholine and lysophosphatidic acid (49). The level of hydrogen sulfide was higher, coupled with weakened detoxification activity for this metabolite in CRC patients compared to healthy individuals (50). Hydrogen sulfide is known for its role in the formation of colon cancer due to induced DNA damage, inflammation of colonic mucosa, mucus synthesis, and DNA methylation (51). Increased bioactive lipids, including polyunsaturated fatty acids, secondary bile acids, and sphingolipids, were detected in patients with advanced colorectal adenomas compared to matched controls (52).

### 3.2 Pancreatic tumor microbiome

Mounting studies documented an emerging role of the pancreatic tumor microbiome in cancer progression and modulating the treatment response (53). *Helicobacter* DNA was detected in pancreatic tumors or surrounding tissue samples but not in controls suggesting the implication of *Helicobacter* spp. in the development of chronic pancreatitis and pancreatic cancer (54). Co-cultivation of pancreatic cells with *Helicobacter pylori* enhanced their malignant potential, leading to a higher activity of NF-κB and AP-1 with consequently dysregulated cellular processes (55). Although *Fusobacterium nucleatum* was described mainly in colorectal tumors, this bacterium was also enriched in breast and pancreatic tumors (8, 56). Mitsuhashi et al. documented the presence of *Fusobacterium* species in 8.8% of pancreatic ductal adenocarcinoma (PDAC) tissues, showing a correlation with shorter survival rates in cancer patients (57). The examination of 113 human PDAC samples detected the presence of bacterial DNA in 76% of tumor samples. According to the findings, the majority of bacteria belong to the *Gammaproteobacteria*, responsible for gemcitabine resistance (58). However, the study by Thomas et al. did not confirm statistically significant differences in the pancreatic microbiome between normal pancreatic tissues compared to tumor samples (59).

In 2018, Pushalkar et al. reported the involvement of the gut and tumor microbiome in PDAC tumor-promoting potential. Bacterial ablation by orally administered antibiotics caused reprogramming of PDAC in mice with a protective effect against tumor progression. The gut microbiome of PDAC patients showed a higher abundance of Proteobacteria, Actinobacteria, Fusobacteria, and Verrucomicrobia compared to healthy controls (60). Interestingly, the analysis of tumor microbiome in PDAC patients showed a potential translocation of Proteobacteria into tumors since the abundance of this bacterial phylum represented 50% in the cancerous tissue (60).

Riquelme et al. reported that prolonged survival of PDAC patients was associated with higher bacterial diversity in the tumor microbiome. The abundance of *Saccharopolyspora*, *Pseudoxanthomonas*, and *Streptomyces* in tumors served as a predictive marker of long-term survivorship with better outcomes. Intratumoral bacteria can modulate the immune tumor microenvironment, whereas the bacterial diversity of the tumor microbiome can contribute to the anti-tumor immune responses with the activation of specific immune cells. Higher levels of Granzyme B+, CD3+, and CD8+ T cells were detected in long-term survivors compared to short-term survivors (61). Guo et al. documented that the basal-like subtype of PDAC was enriched in *Acinetobacter*, *Pseudomonas*, and *Sphingopyxis*. The presence of three bacterial genera positively correlated with DNA replication and the K-ras signaling pathway but negatively correlated with the metabolism of bile acids (62).

Panebianco et al. described that butyrate treatment affected lipidome and metabolome in pancreatic cancer nude BALB/c mice. In addition, butyrate supplementation led to an increase in saturated palmitic acid and stearic acid in gemcitabine-treated mice (63). High expression of aryl hydrocarbon receptor (AhR) correlated with cancer progression. *Lactobacillus* produced tryptophan metabolite active AhR in tumor macrophages. Although, the elimination of dietary tryptophane decreased the function of AhR, leading to the accumulation of TNFα+IFNγ+CD8+ T cells within tumors (64). The animal study showed that high-fat diet-supplemented obese pancreatic tumor-bearing mice did not respond to gemcitabine and paclitaxel in contrast to control diet-supplemented mice. Queuosine-producing bacteria dominated the gut microbiome of obese mice, while S-adenosyl methionine (SAM)-producing bacteria were elevated in control diet-fed mice. Fecal transfer from the normal into obese mice restored the chemotherapy effect to induce oxidative stress and cause cancer cell death (65). Mirji et al. noted that gut metabolite trimethylamine N-oxide (TMAO) might improve the efficacy of immunotherapy in pancreatic cancer patients. Intraperitoneal administration of TMAO reduced tumor growth in pancreatic tumor-bearing mice and led to increased levels of effector immune T cells within the tumor microenvironment. The combined therapy of TMAO and anti-PD-1 improved survival in pancreatic tumor-bearing mice (66).

### 3.3 Gastric tumor microbiome

Infection with *Helicobacter pylori* is a known risk factor for the development of gastric cancer. The presence of this bacterium is associated with changes in stomach acidity leading to differences in bacterial taxa composition (67, 68). Moreover, other biological factors might participate in gastric cancer development by playing the role in the maintenance of the cancerous lesion microenvironment. The data by Chen et al. showed that microbial alpha diversity was higher in tumor samples of patients over 60 years compared to younger participants (69).

Evaluating the gastric microbiome in normal, peritumoral, and tumoral tissues revealed an increased Proteobacteria in peritumoral samples while elevated Firmicutes and Fusobacteria in tumoral specimens. Specifically, an abundance of *Halomonas*, *Shewanella*, *Enterococcus*, *Brevundimonas*, and decreased *Legionella* was detected in peritumoral samples. *Streptococcus, Peptostreptococcus, Lactobacillus, Bifidobacterium, Neisseria, Veillonella*, and *Shewanella* dominated in tumoral samples compared to normal gastric mucosa tissues. According to the findings, immune system downregulation correlated with increased levels of immunosuppressive cells BDCA2+pDCs and Foxp3+Tregs within the tumor microenvironment (70). Li et al. sequenced biopsies from 33 participants with *Helicobacter pylori*-associated chronic gastritis, gastric intestinal metaplasia, gastric adenocarcinoma, and controls. The presence of *Flavobacterium, Klebsiella, Serratia marcescens, Stenotrophomnonas, Achromobacter*, and *Pseudomonas* characterized the tumor samples. In addition, *H. pylori* dominated the samples from *H. pylori*-positive subjects, while *Haemophilus, Serratia, Neisseria*, and *Stenotrophomonas* were more abundant in *H. pylori-*negative samples. The microbial composition of tumor and adjacent non-tumor samples were similar (71).

The analysis of alpha and beta diversity among participants with chronic gastritis and gastric carcinoma showed reduced diversity with dysbiotic potential in gastric carcinomas. Proteobacteria, including *Phyllobacterium, Achromobacter, Xanthomonadaceae*, and *Enterobacteriaceae* dominated the gastric cancer microbiome. Based on the results, *Helicobacter* and *Neisseria* were also present in tumor tissues but at a lower level. Data revealed that a specific nitrosating bacterial community within gastric carcinoma had the genotoxic potential (72). Proteobacteria dominated almost 90% of cancerous samples obtained by subtotal gastrectomy in 62 gastric cancer patients. *Peptostreptococcus, Streptococcus*, and *Fusobacterium* were found in tumor samples. On the other hand, adjacent non-tumor tissue samples contained lactic acid-producing bacteria (69). Specific metabolic pathways, including nucleotide-, energy-, and carbohydrate metabolism were described in cancerous samples [52]. 454 pyrosequencing of the 16S rRNA gene revealed higher nitrate-reducing bacteria in biopsies from cancer patients compared to controls, but the differences were not significant (73).

Wang et al. noted that Actinobacteria, Bacteroides, Firmicutes, Fusobacteria, SR1, and TM7 dominated gastric mucosal biopsies from intraepithelial neoplasia and tumor tissue compared to healthy controls. At the genus level, *Granulicatella, Porphyromonas*, unclassified *Gemellaceae, Rothia*, and *Fusobacterium* were abundant in intraepithelial neoplasia. Further, *Helicobacter* and *Lactobacillus* were increased in tumor tissues. Specific differences in gastric microbial composition might serve as a potential predictor of disease stages (74). In addition to obtained data, an extensive investigation needs to elucidate the emerging role of gastric microbiome and the gastric tumor microenvironment in diagnosis, prevention, and cancer treatment (75).

Dai et al. performed metabolome profiling of gastric tumor samples and matched non-tumor tissue samples. Higher bacterial diversity, together with increased levels of amino acids, carbohydrates, carbohydrate conjugates, glycerophospholipids, and nucleosides, were detected in tumor tissue samples. Several metabolites might be considered as biomarkers for the difference between tumor and non-tumor tissues, including 1-methylnicotinamide and N-acetyl-D-glucosamine-6-phosphate. *Helicobacter* was decreased, while *Lactobacillus*, *Streptococcus*, *Acinetobacter*, *Prevotella*, *Sphingomonas*, *Bacteroides*, *Fusobacterium*, *Comamonas*, *Empedobacter*, and *Faecalibacterium* were enriched in tumor samples. All detected bacteria were associated with metabolome profiling in cancer tissue samples due to different metabolites in the pathway of amino sugar and nucleotide sugar metabolism (76). Metabolites involved in the lipid metabolism and peroxisome proliferator-activated receptor signaling pathway differed between control mice and the model group of N-Methyl-N0-nitro-N-nitrosoguanidine (MNNG)-induced murine gastric precancerous lesions. *Lactobacillus* and *Bifidobacterium* dominated the model group. *Turicibacter*, *Romboutsia*, *Ruminococcaceae*_UCG-014, *Ruminococcaceae*_UCG-005, and *Ruminococcus*_1 decreased in the model group. Metabolites, including N-arachidonoyl tyrosine, cucurbitacin D, phosphatidyl inositols, tryptophylhydroxyproline, and 10-epoxy-12-octadecenoic acid positively correlated with the presence of *Lactobacillus* and *Bifidobacterium*. On the other hand, a negative correlation was observed between ursodeoxycholic acid, β-amyrin, and phenylalanylvaline and those two bacterial genera (77). Yang et al. did not observe significant differences in bacterial diversity between proximal and distal gastric tumor tissues. *Methylobacterium*_ and *Methylorubrum* were elevated in distal tumors. *Rikenellaceae*_RC9_gut_group, *Porphyromonas*, *Catonella, Proteus, Oribacterium*, and *Moraxella* were more prevalent in proximal tumors. Metabolomics showed 30 discriminative metabolites between proximal vs. distal tumors. Purine metabolism, D-glutamine, and D-glutamate metabolism, sphingolipid signaling pathway, taurine, and hypotaurine metabolism, arginine biosynthesis, alanine, aspartate, glutamate metabolism, β-alanine metabolism, butanoate metabolism, ascorbate, and aldarate metabolism, and nicotinate and nicotinamide metabolism were enriched pathways in distal tumors. *Lactobacillus, Muribaculaceae, Rikenellaceae*_RC9_gut_group, and *Morganella* were positively associated with amino acid metabolic pathway and glucose metabolism in proximal tumors (78).

### 3.4 Breast tumor microbiome

Mounting evidence highlights the role of the breast microbiome in women’s health and disease (79). The microbiome analysis of mammary tissues from lumpectomies, mastectomies, or breast reduction revealed that *Bacillus, Acinetobacter, Enterobacteriaceae, Pseudomonas, Staphylococcus, Propionibacterium, Comamonadaceae, Gammaproteobacteria*, and *Prevotella* dominated in the Canadian mammary samples, whereas *Enterobacteriaceae, Staphylococcus, Listeria welshimeri, Propionibacterium*, and *Pseudomonas* were the most abundant in Irish cohort (80).

Microbiota-induced changes in metabolic pathways are associated with the heterogeneity of breast cancer. Modified composition of the gut and breast microbiome promotes the progression of breast cancer (81, 82). In this context, different breast microbiome was observed in women with benign or malignant disease. Breast tissues from women with invasive breast cancer showed an increased abundance of *Fusobacterium, Atopobium, Hydrogenophaga, Gluconacetobacter*, and *Lactobacillus.* Increased cysteine and methionine metabolism, glycosyltransferases, and fatty acid synthesis were observed in benign tissues while cancerous tissues showed reduced inositol phosphate metabolism (79). Xuan and colleagues detected *Methylobacterium radiotolerans* in 100% of analyzed breast tumor tissues whereas *Sphingomonas yanoikuyae* was enriched in paired normal tissues from the same estrogen receptor (ER)-positive breast cancer patient (83). Glycosphingolipid ligands expressed by *Sphingomonas yanoikuyae* activate invariant Natural killer T (iNKT) cells. iNKT cells have a protective role and promote antitumor immunity in regulating breast cancer metastasis (84, 85).

A comparison of healthy vs. cancer-associated breast microbiomes showed that *Bacillus, Staphylococcus, Enterobacteriaceae Comamondaceae*, and *Bacteroidetes* were more abundant in breast tumors from cancer patients while *Prevotella, Lactococcus, Streptococcus, Corynebacterium*, and *Micrococcus* dominated in normal tissues of healthy participants. *Escherichia coli* belonging to the *Enterobacteriaceae* family was more prevalent in cancer patients but without significant differences in microbial composition between breast cancer and adjacent tissues. Further *in vitro* experiments reported a higher level of DNA double-stranded breaks in HeLa cells after exposure to *Escherichia coli* isolates from normal adjacent tissues of breast tumors (86). Hadzega et al. performed RNA-seq analysis of breast tumors and normal tissues from 23 Slovak individuals. In addition, 91 samples obtained from the SRA database and originating in China were included in the study. As results showed, Proteobacteria, Firmicutes, and Actinobacteria were dominant constituents in both cohorts. However, Bacteroides in Slovak samples and Cyanobacteria in Chinese samples were differently abundant (87).

Chiba et al. noted that chemotherapy altered the breast tumor microbiome with a shift in specific microbes. Based on the results, the presence of tumor-specific bacteria might correlate with cancer recurrence (88). Neoadjuvant chemotherapy regimens as a combination of anthracycline, alkylating agents, and taxanes are used to shrink the breast tumor before the surgical procedure (89). Data indicated that neoadjuvant chemotherapy led to an elevated level of *Pseudomonas* and decreased *Prevotella* in breast tumors. The abundance of *Brevundimonas* and *Staphylococcus* was detected in primary tumors from cancer patients who developed the metastatic disease (88). *Pseudomonas aeruginosa* was found in 56% of primary breast tumors and 20% of the normal surrounding mammary tissues. Further analysis showed that *Pseudomonas aeruginosa* modulated cancer cell proliferation and doxorubicin-mediated cell death (88).

More data concerning the role of breast microbiota-derived metabolites in carcinogenesis are warranted. Cadaverine, succinate, and p-cresol might be used as diagnostic markers of breast cancer (90). Wang et al. confirmed that *Clostridiales* and related metabolite TMAO were prevalent in triple-negative breast tumors. Detected levels of plasma TMAO correlated with better response to PD-1 blockade. *In vivo* experiments showed that TMAO can activate endoplasmic reticulum stress kinase, which leads to pyroptosis in cancer cells and elevated anti-cancer immunity *via* CD8+ T cells (91). The gut microbiome is a producer of bile acids. Breast cancer cells are not in direct contact with gut bacteria. However, bacterial metabolites produced by the gut might enter human circulation and then transport to the breast tissue. Production of lithocholic acid was detected as reduced in the early stages of breast cancer (92). Juan et al. showed that probiotic supplementation decreased the occurrence of chemotherapy-related cognitive impairment (CRCI) with modulated plasma metabolites, including p-mentha-1,8-dien-7-ol, linoelaidyl carnitine and 1-aminocyclopropane-1-carboxylic acid in breast cancer patients (Stage I-III) (93).

### 3.5 Lung tumor microbiome

Although the lungs of healthy individuals were considered sterile, new culture-independent methods and advances in molecular analysis helped to reveal the presence of the lung microbiome (94). In healthy lungs, the studies identified a rich and diverse microbiome with a high abundance of *Bacteroidetes, Firmicutes*, and *Proteobacteria*, and the prominent genera *Prevotella, Veillomella, Streptococcus, Neisseria*, *Haemophilus*, and *Fusobacterium* (95–98).

Peters et al. noted that the microbiome of paired normal lung tissues might serve as a biomarker of lung cancer prognosis, presenting a lower diversity in tumor samples compared to paired normal tissues. The results showed that increased levels of *Koribacteraceae* and *Sphingomonadaceae* in paired normal lung tissues correlated with increased survival while the abundance of *Bacteroidaceae, Lachnospiraceae*, and *Ruminococcaceae* indicated reduced survival. However, the composition of the lung cancer microbiome was not associated with recurrence-free or disease-free survival (99). A study concerning the differences in specific squamous cell lung tumor microbiome between smokers and non-smokers revealed a higher abundance of *Acidovorax, Ruminococcus, Oscillospira, Duganella, Ensifer*, and *Rhizobium* in smokers´ samples. As noted, *Acidovorax, Klebsiella, Rhodoferax, Comamonas*, and *Polarmonas* were more prevalent in squamous cell lung tumors with *TP53* mutations, showing an association between the enrichment in selected microbial taxa and *TP53* mutation status (100).

16S rRNA sequencing of lung tumor samples from 89 patients with non-small cell lung cancer (NSCLC) showed a dominance of Actinobacteria, Proteobacteria, Firmicutes, and Bacteroidetes in tumors and adjacent tissue samples. However, no significant differences were observed at the phylum level. In late-stage cancer, the amount of *Pseudomonas*, *Burkholderia*, and *Aquabacterium* was lower while *Corynebacterium, Sphingomonas, Streptococcus, Neisseria, Halomonas, Kocuria, Parvimonas*, and *Rothia* were more presented compared to early-stage tumors (101). Yu et al. revealed that alpha diversity was higher in non-malignant lung samples in contrast to malignant samples. Proteobacteria, Firmicutes, Bacteroidetes, and Actinobacteria were presented in 80% of non-cancerous lung tissues. At the genus level, the results showed the presence of *Acinetobacter, Pseudomonas, Ralstonia*, and two unknown genus-level groups in non-malignant lung tissue samples. *Thermus* was increased, and *Ralstonia* was reduced in adenocarcinomas compared to squamous cell carcinoma samples. According to the findings, *Thermus* levels were higher in lung cancer stages IIIB and IV (102).

Recently, a study on lung cancer patients showed reduced levels of *Corynebacterium, Lachnoanaerobaculum*, and *Halomonas* in lung tumor samples (103). Conversely, a study performed by Apopa and colleagues identified that Actinobacteria, Firmicutes, Cyanobacteria, Acidobacteria, and Chloroflexi were more abundant in lung tumors. Moreover, phyla Bacteriodetes and Proteobacteria dominated in lung cancer samples, with a relative abundance of 57% and 24%, respectively (104). Huang et al. documented the high prevalence of *Veillonella* and *Rothia* and the significant reduction of *Streptococcus* in squamous cell lung carcinoma with distal metastasis compared to samples without metastatic spreading (105). Liu observed that *Streptococcus* was more abundant in lung cancer cases and *Staphylococcus* was highly-presented in lung samples from controls who consented to bronchoscopy examination (106).

Analysis of the salivary microbiome in NSCLC patients revealed a higher abundance of *Veillonella* and *Streptococcus* while decreasing *Bacteroides, Fusobacterium, Prevotella*, and *Faecalibacterium*. Pathways associated with xenobiotics biodegradation and metabolism were overexpressed in cancer patients. On the contrary, folate metabolism-related pathways were downregulated (107). Disrupted gut microbiome in lung cancer is associated with under-represented pathways involved in energy metabolism and ABC-type transport system signaling pathways (108).

Ge et al. described 23 murine microbial metabolites with antiproliferative activity in human lung cancer cells, including Pyrrospirone F, chrysophanol, physcion, and purpuride G. Purpuride G is a newly discovered sesquiterpene lactone with the function to block the cell cycle and induce cell death in lung cancer cells (109). Analysis of gut microbial composition and serum metabolite profiles in 30 lung cancer patients confirmed that L-valine reduced with disease progression. L-valine correlated with the presence of *Lachnospiraceae_*UCG-006 (110). Lee et al. noted that *Enterococcus* and *Lactobacillus* were high in feces from NSCLC patients. The presence of *Bifidobacterium bifidum* was associated with responders to therapy, while *Akkermansia muciniphila* and *Blautia obeum* correlated with non-responders. According to the findings, serum metabolic profile of murine model with anti-PD-1 and *Bifidobactderium bifidum* KCTC3357 was enriched by L-tryptophan, uric acid, and N-acetyl zonisamide (111).

### 3.6 Glioblastoma tumor microbiome

Since the existence of the blood-brain barrier prevents the diffusion of toxic biological materials and bacteria into the brain environment, the brain has been historically considered a sterile organ. However, several studies detected microbial sequences in pathological and non-pathological human brain samples. Massive parallel sequencing of cerebral white matter-derived RNA from HIV/AIDS patients, other disease controls, and surgical resections for epilepsy reported that Proteobacteria was the most abundant phylum in all brain samples. On the other hand, representatives from Firmicutes were undetectable in most brain-derived RNA samples (112). Previous *in vitro* studies documented the internalization properties of gut bacteria in HIV/AIDS patients (113). In the human and mouse brain, the presence of members belonging to Firmicutes, Proteobacteria, and Bacteroidetes were identified under noninfectious or nontraumatic conditions (114).

As shown, specific bacteria play a role in the development of numerous pathologies, including cancer, but the occurrence of bacteria in central nervous system (CNS) tumors has not been widely investigated. A combination of immunohistochemistry, fluorescence *in situ* hybridization (FISH), electron microscopy, 16S rRNA sequencing, and culturomics brought the results describing the tumor-associated microbiota in solid tumors, including glioblastoma (GBM) (8). The microbiome analysis of 40 GBM samples, coupled with the set of negative controls to avoid possible contaminations, detected bacterial DNA in over 40% of the samples. A total of 22 bacterial taxa were identified in GBM tumors by 16S rRNA sequencing. The localization of bacteria mainly within tumor cells was confirmed by a combination of immunohistochemical staining with antibodies against bacterial LPS, lipoteichoic acid (LTA), and 16S rRNA *in situ* hybridization assay (115). Zhao et al. developed a direct 3D quantitative *in situ* imaging of bacterial LPS within gliomas to minimize the risk of contamination by sample collection and processing. A contamination-free manner of the study was achieved by a combination of tissue clearing, immunofluorescent labeling, optical sectioning microscopy, and image processing. Immunohistochemical LPS and LTA staining and 16S rRNA FISH analysis were performed to complement 3D information for tissues from the same samples. According to the findings, bacterial components were predominantly localized in the intercellular space or close to nuclear membranes (116).

Tumor-associated bacteria in GBM raises the question of whether the tumor microbiome is associated with the development of gliomas or if it is the result of damaged blood-brain barrier permeability and glioma-associated immunosuppressive microenvironment. As described, gut microbiota-derived metabolites contribute to the pathogenesis of neurodegenerative diseases *via* disruption of blood-brain barrier integrity, affecting brain functions (117). The emerging evidence of the role of the gut-brain axis in neuro-oncology has been recently reviewed (118). Patrizz et al. documented that glioma development led to a changed gut microbiome in the tumor-bearing murine model. Glioma growth was responsible for an increased presence of the *Akkermansia* genus and Verrucomicrobia phylum in stool samples (119).

The changes in the gut microbiome composition and subsequently altered gut metabolites affect the immune system and CNS immunity (120). Pommepuy et al. evaluated the effect of the fungal metabolite Brefeldin A to induce apoptosis and cell growth inhibition in glioblastoma cell lines. The metabolite was responsible for 60% of cell growth inhibition, and cell cycle arrest in the early G0/G1 phase with a decrease in the S cell population. Based on the data, Brefeldin A was a potential inducer of apoptosis, a modulator of the cell cycle, and might be considered a promising candidate for the treatment of glioblastomas (121). Aerobic abscesses can be differentiated from glioblastomas according to the metabolite ratio. The choline was increased in the enhancing rim of glioblastomas, while creatine and N-acetylaspartate were decreased compared to normal brains. Acetate was not detected in aerobic bacteria abscesses. Lactate and lipid were observed in 8 patients with aerobic abscesses and 5 patients with a tumor (122). Himmelreich et colleagues showed that metabolite profiling was similar between gliomas and *Staphylococcus aureus* abscesses. However, N-acetylaspartate was under-represented, while lipid and lactate, glutamine, and/or glutamate levels were increased in *Staphylococcus aureus* abscesses (123).

### 3.7 Tumor microbiome in gynecological cancers

The role of microbiome in gynecological cancers is documented mainly in studies of vaginal microbiome composition (124, 125). Microbiota influences gynecological carcinogenesis by regulating estrogen levels, immune response modulation, interference with carbohydrate metabolism, and production of microbiota-derived metabolites and bacterial toxins (126).

#### 3.7.1 Ovarian tumor microbiome

Different bacterial composition was identified within cancerous and normal non-cancerous ovarian tissues. The microbiome analysis of ovarian tumor tissues by 16S rRNA sequencing reported a decreased diversity in tumor samples compared to healthy distal fallopian tube samples. A higher level of Proteobacteria was observed in ovarian cancer tissues, whereas Firmicutes and Acidobacteria were down-regulated. At the genus level, *Acinetobacter, Sphingomonas*, and *Methylobacterium* were found in high levels among tumor samples, while *Lactococcus* was significantly reduced (127). Wang et al. detected a decreased amount of *Crenarchaeota* and elevated levels of *Aquificae* and *Planctomycetes* in cancerous samples. At the species level, normal tissues were enriched in *Halobacteroides halobius* (14%), *Gemmata obscuriglobus* (11%), and *Methyloprofundus sedimenti* (10%). The results showed that *Gemmata obscuriglobus* (13%), *Halobacteroides halobius* (11%), and *Methyloprofundus sedimenti* (11%) dominated tumor samples (128). In a study of 99 ovarian cancer samples, 20 matched control samples from non-cancerous adjacent tissue, and 20 unmatched controls found that Proteobacteria and Firmicutes predominated in cancer ovarian samples. In addition, a lower amount of Bacteroidetes, Actinobacteria, Chlamydiae, Fusobacteria, and Tenericutes was detected in cancer samples. As shown, no common bacterium was detected between cancer, pathological noncancerous tumor-adjacent tissue, and non-matched control samples (129).

Nene et al. reported that lactobacilli did not dominate in cervicovaginal microbiome of ovarian cancer patients and *BRCA1/2* mutation carriers younger than 50 years. According to the findings, specific vaginal microbial community with less than 50% of *Lactobacillus* spp. might be causal for ovarian cancer development (130). More recently, a correlation was found between the microbiome composition of the upper reproductive tract and ovarian cancer status. *Acidovorax, Acinetobacter, Aeromonas, Cloacibacterium, Conexibacter, Mariomonas, Methylobacterium, Propionibacterium, Pseudoalteromonas, Vibrio*, and *Xanthomonas* spp. were less presented in upper reproductive tract tissues of ovarian cancer patients, while *Bosea, Mesorhizobium, Mycobacterium, Ralstonia*, and *Variovorax* were more prevalent in cancer tissues compared to control tissues (131). The study concerning the oncobiotic peritoneal microbiome demonstrated that ovarian cancer patients showed a unique peritoneal microbial profile compared to patients with a benign mass. Moreover, 18 highly-specific microbial features were identified by a machine learning algorithm (132).

Antibiotic supplementation affected circulating gut microbes-derived metabolites in mice with injected epithelial ovarian cancer (133). Terrein purified from the fermentation metabolites of *Aspergillus terreus* strain PF26 inhibited the proliferation of ovarian cancer stem-like cells with cell cycle arrest. This studied metabolite might be used as a potential candidate for ovarian cancer treatment (134). Many urinary metabolites were under-represented in ovarian cancer patients. Methanol, with a 65% decrease, was observed in ovarian cancer patients compared to healthy participants. Metabolites, including propylene glycol and mannitol were not changed in healthy and cancer participants (135). In ovarian cancer, diamine oxidase increased in plasma and tumor tissues. Diamine oxidase caused a higher accumulation of produced gamma-aminobutyric acid (136). D’Amico et al. suggested that selected gut microbiome members might affect the level of lactate metabolite. Lactate producers such as *Coriobacteriaceae* and *Bifidobacterium* dominated the gut microbiome of platinum-resistant ovarian cancer patients (137).

#### 3.7.2 Endometrial tumor microbiome

Still, a few studies have focused on endometrial microbiome composition (138). *Lactobacillus* was the most presented genus within the healthy endometrium milieu in most studies. However, some findings reported that other bacterial taxa formed a large part of the microbiome, including *Pseudomonas, Acinetobacter, Vagococcus, Sphingobium* (139), and *Klebsiella pneumoniae, Clostridium botulinum, Pasteurella multocida*, and *Hydrogenophaga* spp. *NH-16* (140).

The association of endometrial *Pelomonas* and *Prevotella* with tumor burden was described among endometrial cancer patients. Additionally, reduced alpha diversity was observed in endometrial samples from cancer patients (141). Lu et al. noted that *Micrococcus* was increased in endometrial microbiome of women with endometrial cancer undergoing hysterectomy while *Pseudoramibacter_Eubacterium, Rhodobacter, Vogesella, Bilophila, Rheinheimera*, and *Megamonas* were more abundant in endometrial microbiome of women with benign uterine lesions (142). Differences in uterine microbiome between patients with endometrial cancer and patients with the benign disease showed that *Porphyromonas somerae* was presented in all patients with Type II endometrial malignancy compared to 57% of patients with endometrial hyperplasia (143). Results from the study on 17 patients with endometrial cancer, 4 patients with endometrial hyperplasia, and 10 patients with benign condition also revealed the link between *Porphyromonas* spp. and endometrial cancer development (144).

Currently, Hawkins et al. presented that selected bacteria might contribute to the pathogenesis of endometrial cancer. *Acidorovax, Bradyrhizobium, Flavobacterium, Hyphomicrobium, Pelomonas*, and *Pseudomonas* differentiated samples from endometrial cancer and benign uterus. A comparison of endometrial tumor microbiome in obese women detected a higher abundance of Firmicutes and Cyanobacteria, and a decrease in genera *Dietzia* and *Geobacillus* in tumors from black women compared to the samples from white patients. Firmicutes were higher in tumors of non-obese white women than in obese white women (145). According to the previous results, Firmicutes and Actinobacteria were more prevalent in endometrioid carcinoma of African-American women than in Caucasian women. Microbial profiles containing a high prevalence of Actinobacteria, Bacteroidetes, Firmicutes, and Proteobacteria in tumor tissues from obese women correlated with the results from a murine model (146). Caselli et al. evaluated the impact of *Atopobium vaginae* and *Porphyromons somerae* on human HEC-1A endometrial adenocarcinoma cells. The results confirmed that co-cultivation with selected bacteria caused the release of inflammatory cytokines and chemokines by endometrial cells (147).

Studies showed the potential involvement of microbiome-produced bacterial toxins and cancer-promoting metabolites in endometrial cancer (148). Metabolite profiling of serum confirmed the lower levels of threonine but increased long-chain fatty acids (LCFA) C16:1, C18:1, C20:1, C20:2, C22:6, C24, and C24:1 in endometrial cancer patients. Levels of C16:1 and C20:2 correlated with *Ruminococcus* spp., *Prevotella* spp., and *Anaerostipes caccae* in cancer patients. The role of C16:1 might be to stimulate endometrial cancer cell proliferation and metastasis *via* mTOR pathways (149). Alauddin et al. analyzed the effect of treatment with Urolithin A (gut-produced microbial metabolite) in the Ishikawa cell line established from an endometrial adenocarcinoma. Treatment with Urolithin A caused changes in cellular properties in tumor cells. Specifically, Urolithin A treatment significantly decreased Rac1 and PAK1 activity and influenced actin polymerization dynamics (150). The presence of Erysipelatoclostridium and its related metabolite ptilosteroid A was associated with the development of grade 2 radiation-induced intestinal injury (RIII) in endometrial cancer patients. The results showed that both might be used as diagnostic biomarkers for grade 2 RIII (151).

### 3.8 The urinary tumor microbiome

In the long-term, the bladder and urine were considered sterile in healthy individuals (152). A novel comprehensive approach found that *Corynebacterium, Staphylococcus*, and *Streptococcus* dominated in the urine of men with or without a biopsy-proven diagnosis of prostate cancer (153). Pearce et al. noted differences in the urinary microbiomes of women with or without urinary incontinence. *Lactobacillus* and *Gardnerella* were the most frequently identified bacterial taxa in both groups (154).

#### 3.8.1 Prostate tumor microbiome

Besides urinary tract microbes from the external environment, the prostate microbiome might be affected by intestinal microbiome. Additionally, surgical procedures could be a source of microbes from the external environment (155). *Mycoplasma* and its bacterial proteins consider to play a role in prostate cancer due to its presence in the tumor microenvironment, but limited data is still available (155, 156).

Most men do not die from prostate cancer due to improved treatment. Death from prostate cancer can be related mainly to metastatic spreading into the pelvic area, spinal cord, bladder, rectum, bone, and brain (157). An early study concerning the presence or absence of bacteria in prostate samples revealed no bacterial DNA in prostate samples from 18 organ donor controls. Bacteria were detected in prostate samples from radical and simple prostatectomy for prostate cancer and benign prostatic hyperplasia, respectively (158). Recently, mounting studies confirmed that the prostate is not a sterile environment (159). Massive ultradeep pyrosequencing identified the differences in the microbial composition between tumor, peritumor, and non-tumor prostate tissue samples. *Staphylococcus* spp. dominated in tumor and peritumor samples. Whereas, *Streptococcus* spp. was presented in non-tumor prostate samples, which supported the idea that bacteria are part of healthy prostate microbiome (160). Another study confirmed that *Escherichia, Propionibacterium, Acinetobacter*, and *Pseudomonas* were abundant in prostate tumors and matched benign tissue samples from 65 Chinese patients who underwent prostatectomy (159). Shannon et al. proposed that *Propionibacterium acnes* as a carcinogen is involved in prostate cancer. *Propionibacterium acnes* has been linked with the development of prostate cancer because of its high presentation in the urinary male tract after puberty where this bacterium can cause infection (161). Alanee et al. also described modifications in the urinary microbiome within patients with prostate cancer compared to controls. Prostate cancer patients exhibited a higher level of *Veillonella, Streptococcus*, and *Bacteroides* with a lower abundance of *Faecalibacterium, lactobacilli*, and *Acinetobacter* (162).

The growth and progression of prostate cancer might be influenced by the gut microbiome and produced bacterial metabolites that indicate the existence of an axis “gut-prostate” (163). Liss et al. reported alternations of folate and arginine pathways. According to the bacterial composition, *Bacteroides* and *Streptococcus* were more abundant in the microbiome of prostate cancer patients (164). In 2021, Matsushita et al. revealed that SCFA-producing bacteria, including *Alistipes* and *Lachnospira*, were more abundant in prostate cancer men compared to non-cancer participants. Metabolic pathways involved in starch and sucrose metabolism, phenylpropanoid biosynthesis, phenylalanine, tyrosine, and tryptophan biosynthesis, cyanoamino acid metabolism, and histidine metabolism were more prevalent in cancer patients (165). A study comparing metabolic profiles in prostate cancer patients vs. healthy subjects found 28 prostate cancer-specific metabolites, including two nucleosides – pseudouridine and uridine (166). *Slackia* sp. strain NATTS, an equol-producing bacterium, was detected in 34% of prostate cancer patients and 24% of healthy participants. Serum level of equol produced from a bacterial metabolite of daidzein correlated with the level of *Slackia* in feces from healthy and prostate cancer patients (167).

#### 3.8.2 Bladder cancer microbiome

The risk of bladder cancer is lower in women compared to men. Different bladder-associated microbial communities might be the reason why the disease affects fewer women (168, 169). The analysis of 32 bladder tumor samples collected during resection/radical cystectomy identified lower levels of Actinobacteria in tumor tissues. Following the findings that higher bacterial diversity indicates healthy microbiome, increased diversity was observed in non-tumor tissues. Tumor-associated mucosa of 13 patients was enriched in *Barnesiella, Parabacteroides, Prevotella, Alistipes*, and *Lachnospiracea_incertae_sedis.* On the other hand, *Staphylococcus* dominated the tumor microbiome of 6 patients. Interestingly, a higher abundance of *Enterococcus* showed in low-grade tumors. Based on these findings, detected microorganisms might play a role in the initial development of bladder cancer (170). Mansour et al. analyzed bacterial community in the urine and bladder carcinoma samples to improve the accuracy of diagnostic in bladder tumor research. According to the findings, *Akkermansia, Bacteroides, Clostridium sensu stricto, Enterobacter*, and *Klebsiella* were more present in tissues compared to the urine samples. Description of changes in bacterial composition during bladder cancer progression might be used as a monitoring tool for the disease (171).

Yumba-Mpanga et colleagues identified 24 urine metabolites specific for bladder cancer compared to healthy individuals. The detected level of gluconic acid was statistically different between healthy participants and bladder cancer patients. This metabolite is associated with the pentose phosphate pathway (166). Uridine was observed in lower abundance in urine samples from bladder cancer patients (172). Mager et al. studied inosine levels in the duodenum, jejunum, and cecum of *Bifidobacterium pseudolongum*-colonized mice. According to the findings, the highest level of inosine was in the duodenum, with a decrease along the intestinal tract. Accordingly, products of inosine, such as xanthine and hypoxanthine, were increased in serum. Orally administered inosine in combination with anti-CTLA-4 and CpG treatment in the bladder tumor-bearing mice led to reduced tumor size and correlated with higher levels of IFN-γ+CD4+ and IFN-γ+CD8+ T cells in the spleen. Gavage of live *Bifidobacterium pseudolongum* led to increased efficacy of anti-CTLA-4 in SPF mice. However, heat-killed bacterium was not able to enhance the immunotherapy due to its inability to produce inosine metabolite (173). In the past, Abdel-Tawab assessed the association between the excretion of tryptophan metabolites and the amount of N-nitrosamine in the urine samples from bilharzial bladder cancer patients. The presence of N-nitrosamines was detected in 45% of control participants and 93% of patients. 64% of bladder cancer patients metabolized the tryptophan abnormally (174). 40 day-saccharin consumption led to an increased level of indican - tryptophan metabolite formed by bacterial action and p-cresol in rats. The development of bladder tumors correlated with greater excretion of indican. As shown, saccharine caused biochemical and physiological disruptions associated with altered electrolytes and microbial amino-acid metabolites, contributing to bladder tumor formation (175).

### 3.9 Melanoma microbiome

Due to existing microbial similarities between humans and pigs, porcine models have been used for the study of microbiome composition in melanoma samples, suggesting a potential therapeutic approach. The level of *Fusobacterium* and *Trueperella* was higher in melanoma samples of the Melanoma-bearing Libechov Minipig (MeLiM) model. In addition, the amount of *Fusobacterium nucleatum* significantly increased in the samples with progressive melanoma compared to animals with a regressive form of the disease (176). Mekadim et al. documented that *Fusobacterium, Trueperella, Staphylococcus, Streptococcus*, and *Bacteroides* dominated in melanoma tissue samples of MeLiM pigs while *Lactobacillus, Clostridium sensu stricto* 1, and *Corynebacterium 1* showed higher abundance in healthy skin samples. Different bacterial diversity was detected in samples collected from specific areas, including melanoma tissue, surface, and healthy skin tissue. A decrease in diversity was observed in melanoma tissues from the MeLiM progressive melanoma group compared to MeLiM regressive animals (177).

Salava et al. noted that *Propionibacterium, Staphylococcus*, and *Corynebacterium* dominated in melanoma samples obtained during surgery using a sterile flocked swab (178). The analysis of bacterial cultures using swab sampling identified the dominance of *Corynebacterium* in 76.9% melanoma of patients with stage III/IV and 28.6% melanoma of patients with stage I/II, respectively (179). Analysis of 17 melanoma metastases revealed HLA-I and HLA-II peptides derived from 41 bacterial species which can induce immune reactivity. 11 recurrent HLA-I-associated peptides were derived from *Fusobacterium nucleatum, Staphylococcus aureus*, and *Staphylococcus capitis*. Microbiota richness was higher in tumors compared to blood samples (180). The findings concerning the relationship between melanoma microbiome and cancer treatment revealed that tumors from metastatic melanoma patients who responded to immune checkpoint inhibitors were enriched in *Clostridium* (8). Accordingly, Zhu et al. detected an enrichment of *Lachnoclostridium* within cutaneous melanoma of patients with improved overall survival. Higher amounts of *Lachnoclostridium* positively correlated with the abundance of infiltrating CD8+ T cells, which brought benefits or improved outcomes for patients (181).

Frankel et al. reported that melanoma patient response to immunotherapy correlated with gut microbial composition, metabolic profile, previous antibiotic supplementation, and patient diet and lifestyle. A higher level of anacardic acid was observed in feces from responding metastatic melanoma patients to immunotherapy. Bacterial enzymes, which played a role in fatty acid synthesis and inositol phosphate metabolism, dominated treatment responders. In total, changes in 83 metabolites were detected between responders vs. patients with progressive disease (182). According to Coutzac et al., lower amounts of baseline butyrate and propionate correlated with longer progression-free survival (PFS) in patients with metastatic melanoma treated with ipilimumab (183). The role of indole-3-carboxaldehyde (3-IAld) is to maintain normal gut epithelial integrity. Renga et al. confirmed the effect of 3-IAld to protect the gut barrier in melanoma tumor-bearing mice with immunotherapy-induced colitis. Administered 3-IAld helped to improve survival rate, maintain murine weight and protect against developed colitis due to reduced therapy-induced gut damage and toxicity (184). Anthraquinone derivatives termstrin A, B, C, and D were isolated from the *Streptomyces* sp. BYF63. Termstrin B showed a cytotoxic function against melanoma cell line A375 (185). 3,5-dihydroxy Q1 -4-ethyl-trans-stilbene (DETS) is a known bioactive bacterial secondary metabolite isolated from *Bacillus cereus*. DETS helped to downregulate pathways involved in melanoma progression in cells treated with DETS. However, *in vivo* studies that will confirm the antioxidant and anti-cancer properties of DETS are needed (186).

**Figure 2:**
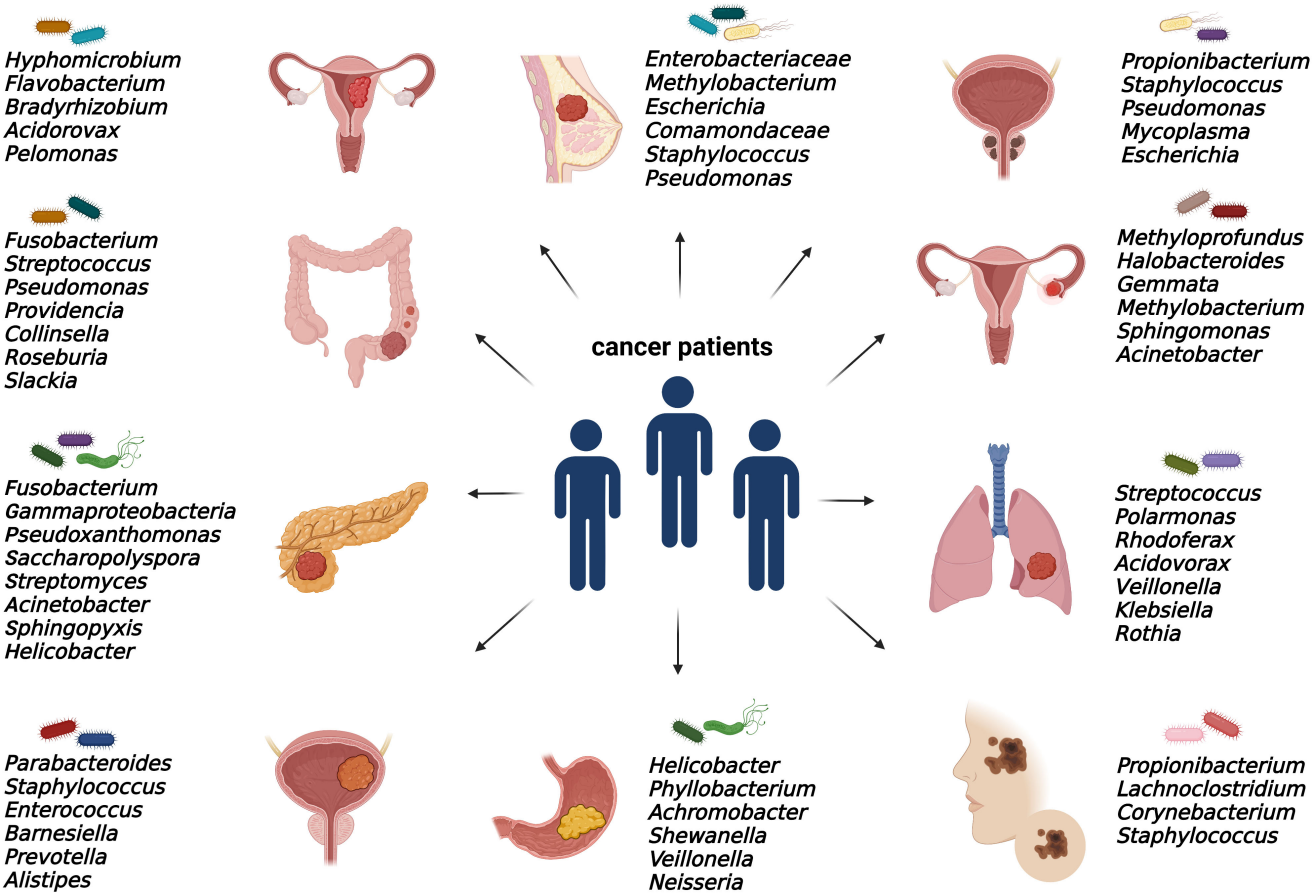
Specific intratumoral bacteria within different types of tumors. The presence of cancer type-specific bacteria within the tumor microenvironment was revealed mainly by 16S rRNA or metagenomic sequencing and *via* immunohistochemical and immunofluorescence detection methods.

As discussed, elucidating the emerging role of microbes within tumors is still an issue of intensive research. Bacterial communities were found in numerous solid malignancies, mainly in ovarian, endometrial, prostate, bladder, breast, pancreatic, colorectal, gastric, melanoma, and lung cancer (**Figure 2**).

## 4 The clinical utility of tumor microbiome-based approach

The results from preclinical models and clinical studies reported significant differences in microbiome composition between tumor samples and healthy tissues. Tumor-associated microbiota contributes to carcinogenesis by activation of oncogenic signaling pathways, such as Wnt/β-catenin signaling. The studies demonstrated that *Helicobacter pylori*, *Fusobacterium nucleatum*, and enterotoxigenic *Bacteroides fragilis* secrete cytotoxin-associated gene A (CagA) protein (187), adhesin A (FadA) (188) and toxin Bft (189), respectively. In addition, intratumoral microbiota inhibits innate and adaptive immune responses (190). A comprehensive approach identified unique microbial cancer-related signatures in tissues and blood (7) by analyzing genome and whole transcriptome sequencing studies from 33 cancer types within The Cancer Genome Atlas (191). This raises the possibility of using tumor-associated microbial biomarkers in cancer screening and diagnostics.

Specific intratumoral bacteria correlated with prognostic clinicopathologic features and metastatic potential. One of the largest studies to date evaluated the role of breast microbiome in fresh-frozen surgical specimens from 221 patients with breast cancer, 18 individuals predisposed to breast cancer, and 69 controls. Microbiome analysis using 16S rRNA sequencing, identified significant differences in the relative abundance of specific bacterial taxa regarding tissue type, cancer stage, grade, histologic subtype, receptor status, lymphovascular invasion, and node-positive status (192). For instance, *Pseudomonas, Proteus, Porphyromonas*, and *Azomonas* were enriched in tumor samples, while a higher abundance of *Propionibacterium* and *Staphylococcus* was observed in healthy control, high-risk, and tumor-adjacent normal tissues. Regarding the association between bacterial genera and prognostic breast tumor features, *Porphyromonas*, *Lacibacter*, *Ezakiella*, and *Fusobacterium* were related to higher stage tumors and lower levels of *Alkanindiges, Micrococcus, Caulobacter, Proteus, Brevibacillus, Kocuria*, and P*arasediminibacterium* were linked to estrogen receptor (ER)-positive status (192). Primary breast tumors from patients who developed distant metastases displayed an increased tumoral abundance of *Brevundimonas* and *Staphylococcus* (88).

According to the findings, the *Fusobacterium nucleatum* gDNA and abundant levels Gal-GalNAc were found not only in colorectal but also in breast cancer tissue samples. Parhi and colleagues noted that fusobacterial colonization is associated with accelerated tumor growth and metastatic progression of breast cancer (193). In a very recent study on murine breast-tumor model MMTV-PyMT, a depletion of intratumoral bacteria was significantly related to the reduction of lung metastasis. As shown, intracellular microbiota reorganized the actin cytoskeleton network within tumor cells, leading to increased survival during breast cancer metastatic spreading (194). Moreover, the metastatic potential of breast carcinomas was increased in tumor models after intratumoral administration of specific genera, isolated from tumor-associated bacteria (194).

Noticeably, several studies described the crucial impact of tumor microbiome on the response to cancer treatment modalities. As noted by Nejman and colleagues, *Gardnerella vaginalis* was more abundant in tumor samples from non-responders (8). In CRC patients, modulation of the innate immune system by *Fusobacterium nucleatum* is considered to play a role in chemoresistance. A preclinical study found a link between *Fusobacterium-*mediated resistance to chemotherapeutics (oxaliplatin and 5-fluorouracil) and autophagy modulation *via* the TLR4 and MYD88 signaling pathway (195). Pancreatic cancer tissues from PDAC samples showed an abundance of intratumoral *Enterobacteriaceae* and *Pseudomonadaceae* families (58). As shown, *Gammaproteobacteria* were able to enzymatically inactivate gemcitabine by expressing the bacterial cytidine deaminase, and antibiotic treatment with ciprofloxacin overcame the gemcitabine resistance (58). The presence of *Mycoplasma hyorhinis* in the TME suggested decreasing the cytostatic activity of gemcitabine *Mycoplasma*-infected tumor cell lines (196).

## 5 Conclusions, limitations and future directions

In conclusion, targeting the gut and tumor microbiome represents an emerging trend in cancer development and treatment. While the associations between intestinal microbiome, carcinogenesis and the response to anti-cancer therapies have been intensively studied, the impact of tumor microbiome requires further investigation. Distinct microbial communities reside within TME, and tumor-associated microbiota showed having tumor-specific character. Mounting evidence from solid cancers described differences in microbiome composition between cancerous and non-cancerous tissues. Recently, studies reported the correlation between the tumor microbiome and the clinical features of corresponding tumors. Further clinical research focusing on the impact of intratumoral bacteria and microbiota-derived metabolites on therapeutic outcomes is highly warranted. A deep understanding of complex interactions between tumor microbiome, cancer cells and TME components might bring clinically relevant information. In addition, comprehensive studies on large cohorts of tumor samples may identify microbial biomarkers predicting metastatic spreading. Precise characterization of tumor microbiome signatures may be used to stratify cancer patients leading to the development of more effective, individualized, tumor-specific therapies. Importantly, the progress in machine learning algorithms can help to uncover the underlying mechanisms and signaling networks. This could lead to identification of potentially novel targets for predicting the treatment response. However, the considerable variation in microbiome composition between patients, the existence of confounding factors, and heterogeneity in host genetic susceptibility, should be carefully considered.

Although an increasing number of microbiome studies, limits in taxonomic resolutions represent an important issue for proper understanding of functional impact of the microbiome. In this context, more comprehensive approach, including metagenomics, metatrascriptomics, metabolomics, and metaproteomics, should more precisely evaluate the role of the microbiome in cancer. One of the most serious concerns in terms of tumor microbiome studies is the risk of microbial contamination during sample collection, storage, and processing. In the future, procedures controlling the development of 3D imaging methods may allow the analysis of direct interactions between the microbial community and other components of host TME. Recent studies focus mainly on relative abundances of bacterial taxa in each sample. This represents a kind of limitation since changes in total abundance might bring more accurate information about the real impact of microbiota and microbiota-derived metabolites on host physiology. In addition, present data describes the occurrence of intratumoral bacteria but does not distinguish whether their presence is causal in terms of cancer development or whether they co-exist in TME due to leaky vasculature and immunosuppressed microenvironment.

Importantly, the co-cultivation of specific bacterial taxa with organoids prepared from pluripotent stem cells or tissues may represent an effective tool to study host–bacteria interactions and evaluate the potential mechanisms behind them. The existence of crosstalk between intratumor and gut microbiomes brings the possibility of modulation-based therapies, including prebiotic and probiotic administration, fecal microbiota transplantation, and others. Thus, personalized determination of patient gut and tumor microbiome composition might represent a potential diagnostic and prognostic tool, and restoration of balance in microbial community may improve treatment efficacy and patient outcomes.

## Author contributions

SC designed the review and prepared the original draft. AS and SC analyzed the literature and wrote the manuscript. SC reviewed and edited the manuscript. AS, SC, and VS prepared the Figures and Table. MM reviewed the manuscript before submission. All authors contributed to the article and approved the submitted version.

## Funding

This study was supported by the Scientific Grant Agency of the Ministry of Education, Science, Research and Sport of the Slovak Republic and Slovak Academy of Sciences (VEGA), contract No. 2/0069/22. The funding source had no influence on the writing of the manuscript.

## Acknowledgments

We would greatly thank Joe Gracik for reading the manuscript carefully and helping with language editing. **Figures 1** and **2** were created with BioRender.com.

## Conflict of interest

The authors declare that the research was conducted in the absence of any commercial or financial relationships that could be construed as a potential conflict of interest.

## Publisher’s note

All claims expressed in this article are solely those of the authors and do not necessarily represent those of their affiliated organizations, or those of the publisher, the editors and the reviewers. Any product that may be evaluated in this article, or claim that may be made by its manufacturer, is not guaranteed or endorsed by the publisher.
